# Socio-Cultural and Somatic Factors Associated with Children’s Motor Competence

**DOI:** 10.3390/jfmk6020054

**Published:** 2021-06-21

**Authors:** Vitor P. Lopes, Diogo Monteiro

**Affiliations:** 1 Instituto Politécnico de Bragança, Campus de Santa Apolónia, Research Center in Sports Sciences Health Sciences and Human Development (CIDESD), 5300-223 Bragança, Portugal; 2 ESECS-Polytechnic of Leiria and Research Center in Sports Sciences, Health Sciences and Human Development (CIDESD), 2411-901 Leiria, Portugal; diogo.monteiro@ipleiria.pt

**Keywords:** motor skills, motor development, biosocial factors, parental attitudes, ecological model

## Abstract

The purpose of this study was to assess the effect of somatic and socio-cultural factors on children’s motor competence (MC). MC was assessed through the standing long jump (SLJ), distance throw of a tennis ball (TTB), and 20 m dash (20 m) in 181 children (84 girls) with a mean age of 6.10(0.47) years. The effect of socio-economic status, house/living space, educational practices, the child’s interaction with peers and siblings, and the sum of five skinfolds (SS) were analysed via structural equation modelling (SEM) in each motor skill. The SEM models displayed a good fit to the data. In addition, standardized direct effects are significant on different outcome variables, except for brotherhood relationship (BR) and peer relationship in TTB, and 20 m dash and BR in standing long jump (SLJ). SS are negatively related to all motor skills.

## 1. Introduction

Motor competence (MC), defined as a person’s proficiency to execute motor skills as well as the underlying mechanisms including motor coordination and control [1,2,3], is associated with health-related behaviours and attributes such as physical activity (PA) and body mass index (BMI) [4,5]. Cross-sectional and longitudinal data both suggest that MC may be important for promoting many aspects of health-related behaviours [3]. Empirical evidence supports associations between MC and a range of health outcomes. Children (7 to 14 years old) with low levels of motor competence tend to have lower levels of physical activity and cardiorespiratory fitness [6]. Lower levels of MC are associated with sedentary behaviours in children 9 to 10 years of age [7]. Higher MC attenuates the decline in physical activity levels [4], and lower MC is associated with increased BMI [5] throughout childhood. MC in childhood is also associated with higher physical activity levels and fitness in adolescence [8,9].

Despite the health benefits associated with MC, the prevalence of children classified as having poor MC has increased in recent decades [10]. It was shown in a European study [11] that around 20% of children are ‘at-risk’ of delays in motor development. In the USA [12] the proportion of children in this category is 70%. Another study comparing children (six to nine years of age) from northern, central, and southern European regions found that most children in each of the regions were rated as typically developing. However, only 0.9% of southern European children who were rated as typically developing scored above the typical range, and were mostly rated as having poor (29.3%) or impaired MC (10.3%).

The identification of correlates and potential mechanisms of change of MC could be crucial to develop interventions targeted to improve children’s MC. Despite the fact that interventions can improve MC [13,14], it remains unclear which correlates should be targeted to ensure interventions are optimized.

Bronfenbrenner [15] proposed the ecological model of human development to explain how the characteristics of children and their environments interact to influence how they grow and develop. The theory emphasizes the importance of studying children in multiple environments, to understand their development. The study of the socio-cultural factors associated with the development of MC has recently received great attention [16,17,18]. In early reviews [19,20,21], associations between specific fundamental skills and parental attitudes, parent–child and sibling interactions were reported. More recently, Malina, et al. [22] claimed the need to study MC development within a bio-cultural approach.

Parents from different socio-economic statuses (SES) tend to have different attitudes towards their children about education, and the process of socialization (the type of learning to which the child is exposed, the way personality and attitudes are developed, the child’s self-concept and behaviour) is related to SES [23]. While it is well established that overweight/obesity (BMI) is negatively correlated with MC [24,25], little is known about socio-cultural factors. Among Australian youth [6], it was found that low MC was associated with low SES in girls. Among boys, there was a strong association between low MC and the likelihood of being from non–English-speaking cultural backgrounds. According to a systematic review [26], a higher socio-economic background was consistently correlated with MC. Nevertheless, socio-cultural correlates of MC development, such as educational practices within the family, siblings’ effects, peer relations, have not received detailed attention in the context of MC development.

Therefore, the purpose of this study was to assess the effect of somatic (body mass index and skinfolds) and selected socio-cultural factors on MC of five to six year old children. Specifically, we hypothesize that excess body fat is negatively associated with MC; and that socio-economic factors, educational practice within the family, interactions with peers, variables related with house/living space, and sibling characteristics/brotherhood relationship, are all determinants factors of MC.

## 2. Materials and Methods

### 2.1. Participants

Participants were *n* = 181 children of both sexes (girls *n* = 84, 46.4%) with mean age of 6.10(0.47) years in an age range between 5 and 6 years (the age year was considered between 0 and 11 months of the respective year), corresponding respectively to 40.3 and 59.7% of the total sample. About half of the children were in kindergarten, while the other half were first graders. Children were recruited as a convenience sample in four urban schools in the inland of the north of Portugal. Not having physical or mental disabilities were the inclusion criteria. Permission was obtained from the respective school director, parents or guardians gave informed consent, and children assented. The ethics committee of the institution of the first author approved this study (Proc. *ec120345*, 9/03/2019).

The tests were administered during two days in each of the children’s schools. Anthropometric measurements and MC assessment were performed on the first day; on the second day, MC tests were performed, in the following sequence: standing long jump, tennis ball throw for distance, and speed run. Between tests, there was at least 5 min of rest. The subsequent test was only performed after the participant indicated that he/she was not tired. All the test and measurements were done by one of the authors.

To estimate the reliability, MC tests were performed a second time, a week later, in 40 participants (20 boys and 20 girls).

### 2.2. Motor Competence Assessment

Motor competence was assessed as the performance (product-oriented assessment) on tennis ball throw for distance, speed run, and standing long jump:

- Tennis ball throw for distance. The test measured how far (aerially) the child could throw a tennis ball within a large, prescribed area using any one-handed throwing pattern. The child could take one step forward during the throw, but was not permitted to step over the restraining line. The child was required to perform seven trials. The final score was the average of five trials, after removing the best and worst throws.
- Speed run. The child ran as fast as possible for 15 m, after a 2.5 m running start. The score was the average of the best two out of three trials.
- Standing long jump. The child jumped horizontally as far as possible, using a two-foot take-off and landing. The score was the average of the best four out of five trials.

Test–retest reliability estimated with intra-class correlation coefficient for this study was 0.60 for speed run, 0.95 for standing long jump, and 0.89 for tennis ball throw for distance.

### 2.3. Socio-Demographic and Environment Variables

Socio-demographic and environment variables were grouped in five main areas:

- Socio-economic status (SES) (level of education and professional occupation of both mother and father, per capita household income);
- Variables related to house/living space (HS) (type of housing e.g., villa, single storey house, apartment in housing block or apartment in detached house with two floors, number of rooms in the house, number of persons per room, existence of a courtyard near the house, terrace, garden, or yard where the child can play);
- Variables that characterize educational practices within the family (EP) (regular father and mother’s presence at home and amount of time out of the home, relative time spent by each parent with the child, geographical limit of children’s play in relation to the household e.g., only at home; at courtyard, terrace, garden, or yard; outside but only in the city block; outside but within the neighbourhood boundaries; doesn’t have any restrictions; type of toys the child owns and uses most often e.g., physically active or passive toys);
- Variables that characterize the child’s interaction with peers (PR) (interaction with other children, not including sisters or brothers, out of school (yes or no), sex of preferred playmates i.e., same sex, opposite sex, age of preferred playmates i.e., same age, younger or older);
- Variables related to sibling characteristics or brotherhood relationship (BR) i.e., number of siblings, birth order, having an older brother(s) and/or older sister(s) (over four years).

### 2.4. Body Dimensions

Five skinfolds (triceps, subscapular, suprailiac, abdominal, and medial calf) were measured to the nearest 0.1 mm using a Holtain skinfold caliper (GPM-caliper, Zurich, Switzerland). The five skinfolds were summed (SKF) to provide an indicator of subcutaneous adiposity.

All skinfolds were measured twice; the average was used for analysis. All measures were done by the same trained technician following a standardized protocol [27]. Technical errors of measurement were between 1.01 mm and 1.75 mm for skinfolds.

### 2.5. Statistical Analysis

Descriptive statistics, including mean and standard deviations were calculated for all studied variables. A structural equation modelling (SEM) via maximum likelihood estimator method was employed in Amos 23.0 to test the model fit, which was performed for each motor skill (the outcome variable). Nominal variables were previously criterion scaled. In addition, standardized direct effects on the outcome’s variables were analysed. Therefore, Bootstrap resampling (1000 samples), via bias corrected 95% confidence intervals (CI) was used to assess the significance of the direct effects. The magnitude of effects was evaluated through Cohen [28] suggestions: 0.20 (small effect size); 0.50 (medium effect size); 0.80 (large effect size). The traditional incremental and absolute goodness-of-fit indexes: Comparative Fit Index (CFI); Tucker–Lewis Index (TLI); Standardized Root Mean Square Residual and Root Mean Square Error of Approximation (RMSEA), and its respective confidence interval (90%) were employed to test the model fit for both CFA and SEM with the cut-off values: CFI and TLI ≥ 0.90; SRMR and RMSEA ≤ 0.08, being assumed.

## 3. Results

A preliminary analysis revealed no outliers and missing values detected. However, skewness and kurtosis values are comprised within cut-off values (all of the are comprising between −2 to +2 for skewness and between −7 to + 7 for kurtosis) revealing no violation from univariate data. Nevertheless, Mardia’s coefficient for multivariate kurtosis exceeds the recommended value (26.12; 24.72; 33.11) for model 1, 2, and 3 respectively. Therefore, a Bollen–Stine bootstrapp (2000) was performed for further analysis. Finally, the collinearity diagnosis was checked via variance inflation factor (VIF), and assuming values less than 10 for VIF and greater than 0.01 for tolerance tests. Therefore, the results showed that both in VIF and tolerance tests scores were below 10 and above 0.1 respectively, ensuring the appropriate conditions to test the regression model.

Since the nominal variables were criterion scaled before entering in the SEM models, it is necessary to know the mean of the criterion (dependent variable) of the participants in each category of the nominal observed variables to interpret structural coefficients. These means are presented in Table 1.

**Figure 1:**
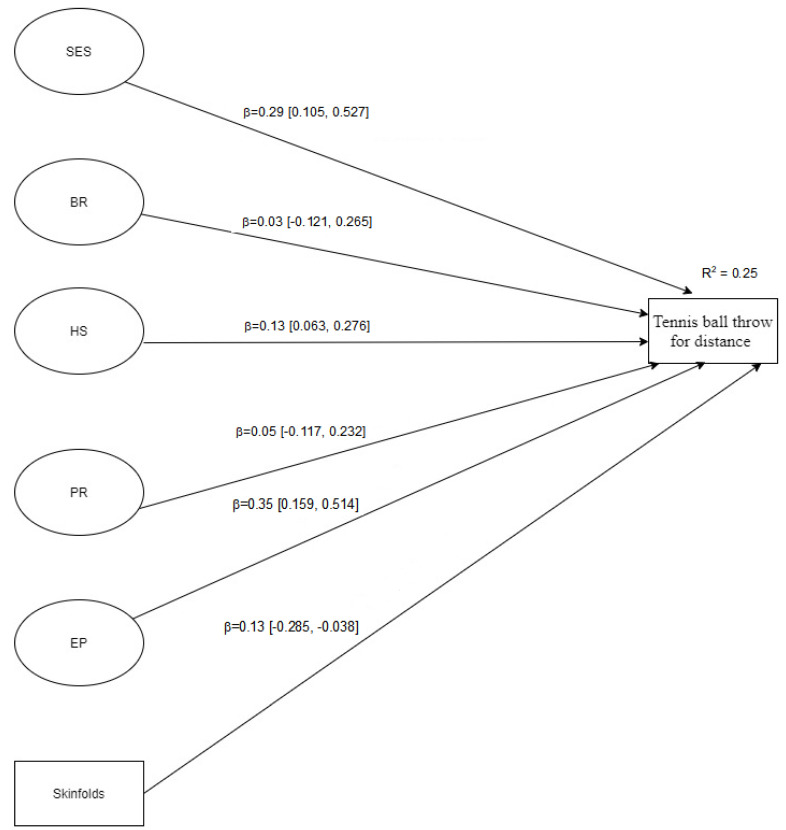
Structural model for tennis ball throw for distance. Note. SES = socio-economic status; BR = brotherhood relationship; HS = house space; PR = peer relationships; EP = educational practice; skinfolds = sum of 5 different skinfolds, including bi-acromial breadth, hip breadth, upper arm girth, standing calf girth, and upper limb length.

**Figure 2:**
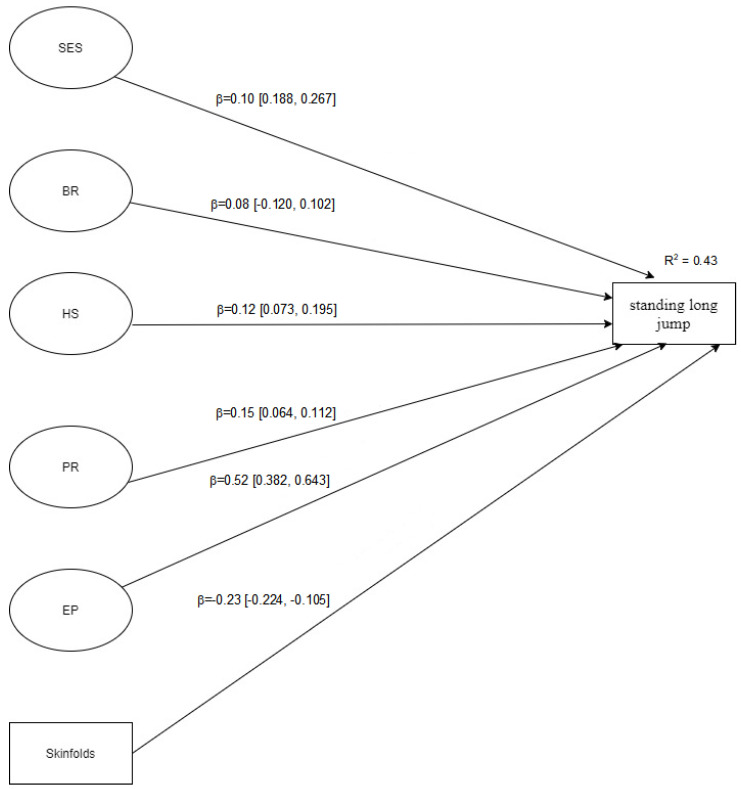
Structural model for standing long jump. Note. SES = socio-economic status; BR = brotherhood relationship; HS = house space; PR = peer relationships; EP = educational practice; skinfolds = sum of 5 different skinfolds, including bi-acromial breadth, hip breadth, upper arm girth, standing calf girth, and upper limb length.

**Figure 3:**
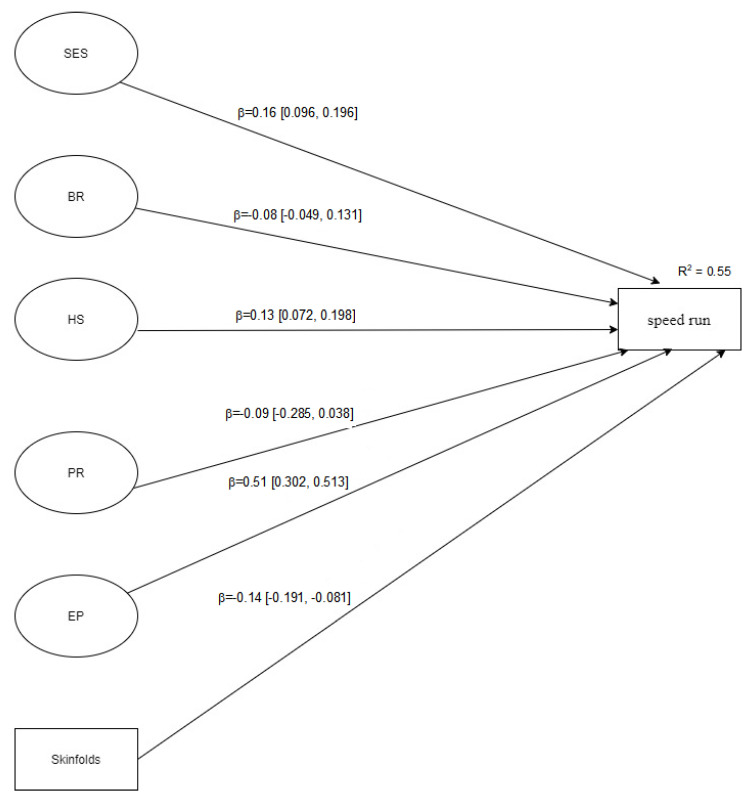
Structural model for speed run. Note. SES = socio-economic status; BR = brotherhood relationship; HS = house space; PR = peer relationships; EP = educational practice; skinfolds = sum of 5 different skinfolds, including bi-acromial breadth, hip breadth, upper arm girth, standing calf girth, and upper limb length.

Figure 1, Figure 2 and Figure 3 showed the structural models analysed considering each motor ability tennis ball throw for distance, standing long jump, and speed run, respectively. The latent variables in each model (Figure 1, Figure 2 and Figure 3) are represented by the variables in Table 1. Overall, all SEM models displayed a good fit to the data, namely: Model 1 (tennis ball throw for distance): [X^2^ (115) = 144.53; SRMR = 0.080; B-Sp = 0.033; RMSEA = 0.038 (90%CI = 0.012, 0.056); TLI = 0.919; CFI = 0.931]; Model 2 (standing long jump): [X^2^ (115) = 165.21; SRMR = 0.065; B-Sp = 0.002; RMSEA = 0.049 (90%CI = 0.031, 0.065); TLI = 0.910; CFI = 0.940]; Model 3 (speed run): [X^2^ (115) = 163.41; SRMR = 0.071; B-Sp = 0.002; RMSEA = 0.048 (90%CI = 0.030, 0.065); TLI = 0.921; CFI = 0.949]. In addition, standardized direct effects are significant on different outcome variables, except brotherhood relationship (BR) in all outcome variables and peer relationship (PR) in tennis ball throw for distance and speed run. The observed effects varied from small to medium.

Adiposity levels (sum of skinfolds) are negatively related to all motor skills performance.

In the SES case, the highest motor performance in the three motor skills was attained by children with parents in the lowest and intermediate levels of education and professional occupation. In the case of house/living space, (HS) the best performance was achieved by children that live in a villa or single storey house and by children that live in a house with a courtyard, terrace, garden, or yard. In terms of peer relationship (PR), the highest performance was obtained by children that interact with others out of the school, when the playmates are older, and of the opposite sex. In the case of educational practices within the family (EP), children that can play in the neighbourhood, whose mother spent more time with them and those that prefer to play with active toys, achieved the best performance.

## 4. Discussion

The aim of the present study was to assess the relationships of socio-cultural (socio-economic status, sibling characteristics/brotherhood relationship, house/living space, child’s interaction with peers, and educational practices within the family) and of body fat with MC in Portuguese children five to six years old in both sexes.

Overall, the formulated hypotheses were confirmed. In fact, socio-economic status (professional occupation and education levels of mother and father), house living space (the type of housing; existence near the house of a courtyard, terrace, garden, or yard where the child can play), the child’s interaction with peers (age of preferred playmates, sex of preferred playmates, interaction with peers out of school), and the educational practices within the family (geographical limit of child’s play in relation to household, type of toys, relative time spent by each parent with the child), are significantly associated with children’s MC. In terms of socio-cultural variables, only sibling characteristics/brotherhood relationship (having an older brother(s), having an older sister(s), birth order) were not significantly associated with MC.

The results of the present study are in line with the results of previous studies. For instance, Zeng, et al. [29] found that parent education and home physical activity environment were significant and positively associated with locomotor skills evaluated with the Bruininks–Oseretsky test of motor proficiency, 2nd edition. Luz, Valente-dos-Santos, Luz, Sousa-e-Silva, Duarte, Machado-Rodrigues, Seabra, Santos, Cumming and Coelho-e-Silva [16] also found that the mother’s educational level was a predictor of MC.

In addition, body fat (sum of skinfolds) was, as expected, negatively associated with MC. Excessive body fat is a negative factor in the development of MC during childhood. This outcome is well established in the literature, both in cross-sectional [25,26,30] and longitudinal studies [30,31,32,33].

Socio-economic factors, such as parent’s occupation and education levels, are factors that are systematically found in the literature to be associated with motor development and MC levels [17,23,31,34,35]. Socio-economic factors such as parents’ education level and parents’ occupational prestige tend to be associated with parenting, namely with parents’ education practices. Child rearing practices vary across social class and ethnic group [36]. Generally, lower socio-economic status is associated with more permissive educational practices, that is, less control over the child’s free time and play activities. Intermediate and higher socio-economic status are usually more associated with restrictive educational practices, that is, they tend to exert more control over the child’s free time and play activities [37]. However, the relationship between socio-economic status and educational practices may culturally differ from country to country, and should not be viewed as stable since they tend to change over time and even decades [15,34]. In the present study, we found that better MC was associated with lower parents’ education and occupational levels, and children that are allowed to play outside in the neighbourhood, those whose mother spent more time with them and that prefer to play with active toys, achieved the best performance. Similar results were found by Antunes, et al. [35]. Inversely, Zeng, Johnson, Boles and Bellows [29] found that parent education was positively associated with locomotor skills, and Klein, et al. [38] found that students with a higher SES exhibited higher motor performance compared to that of lower SES, while Birnbaum, Geyer, Kirchberg, Beulshausen, Manios, Koletzko and On behalf of the ToyBox-study [17] found no significant associations of SES with motor abilities.

In the context of sibling characteristics, we found that it was not associated with MC, which is a divergent result from the literature. Siblings are seen as an essential component of family systems and as a significant context for development and learning [39,40]. Siblings demonstrate the ability to teach one another during home interactions [39,41]. The age differences between siblings mean that two children are likely to have different experiences in the family. First-born children engage in leadership, teaching, and helping roles, whereas second-born children are more likely to imitate, to follow, and be a learner [39,41], having the benefit of learning from an older sibling, leading sometimes to a faster development for second-born children [42,43].

The peer group plays an important role in child socialization, which becomes more important with age. Peers can provide reinforcements and important models for the practice of physical activity, including games and play, and the development of MC [23]. We found that the highest motor skill performance was attained by children that interact with others out of the school environment. In consonance with the present research, Lehto, et al. [44] found that the levels of children’s physical activity tended to be lower if they had weaker social contacts with their peers, and physically active children sought each other’s company [44]. Additionally, we found that if playmates were older and of opposite sex, children had better motor skill performance.

Socio-spatial factors are, according to Klein and Liesenhoff [45], of great importance for development during childhood. As a result of the organization and structure of space, a selective determination of activities and social relations are possible. The existence of certain usable space determines whether it can or cannot be used and whether it permits social contacts. The primary child’s living space is his home and its surrounding area. To what extent living space is favourable or unfavourable for children’s MC development can be found by studying the features of the home: the number of rooms, the size of the rooms, the number of people per room, the number of floors of the house, and the outside space availableKlein and Liesenhoff [45]. We found that the best motor skill performance was achieved by children that live in a villa or single storey house and by children that live in a house with a courtyard, terrace, garden, or yard.

The present study is distinctive in the way that it simultaneously examined the extent to which somatic factors and socio-cultural factors may contribute to children’s MC. The use of SEM, instead of using univariate analysis such as ANVOVA, or even multiple regression, was a major strength since this statistical approach allows for analysis of the effect of sets of variables on MC rather than each variable by itself. However, there are some limitations: the cross-sectional design only permits association inference and not causal inference, as well as the limited sample size and the lack of representability of the convenience sample being possible. Nevertheless, results appeared generally consistent with other studies [17,23,31,34,35]. The present research was based on understanding that individual development requires the analysis of an individual’s experience, and development cannot be separated from the social context [46]. In this framework theory, besides somatic variables, we assessed 21 environmental factors combined to approximate an ecological model containing five contextual subsystems (socio-economic, house/living space, sibling relationships, household, peers, and family education practices).

In conclusion, body fat with a negative effect, socio-economic variables, sibling characteristics/brotherhood relationship, type of housing/living space, child’s interaction with peers, and educational practices within the family, adequately explain the variance in MC in children aged five to six years.

## Figures and Tables

**Table 1:** Mean and standard deviation in each category of nominal predictor variables of each motor skill performance and in sum of skinfolds.

Variables/Categories	Tennis Ball Throw for Distance (m)		Standing Long Jump (cm)		Speed Run (s)	
	M	SD	M	SD	M	SD
SES						
LME						
1 (lowest level)	9.47	4.55	74.50	4.38	3.44	0.03
2	8.81	3.97	88.18	19.49	3.50	0.25
3	9.01	3.08	91.59	17.51	3.49	0.33
4	6.77	1.98	88.81	14.86	3.55	0.33
5 (highest level)	6.95	2.71	84.29	13.40	3.64	0.33
MO						
1 (highest level)	6.86	2.62	84.23	14.30	3.65	0.33
2	6.80	2.51	91.48	11.85	3.57	0.35
3	8.46	3.35	88.83	18.50	3.48	0.29
4 (lowest level	8.56	3.14	85.44	13.12	3.58	0.20
LFE						
1 (lowest level)	9.88	2.19	73.80	5.37	3.60	0.23
2	8.28	3.51	86.45	18.81	3.56	0.31
3	8.74	3.29	99.06	13.89	3.43	0.26
4	7.09	2.61	86.01	14.49	3.59	0.31
5 (highest level)	6.84	2.69	82.89	12.13	3.58	0.38
FO						
1 (highest level)	6.86	2.58	85.56	13.24	3.54	0.38
2	6.87	2.52	86.91	12.73	3.62	0.32
3	7.74	3.14	88.44	16.46	3.53	0.32
4 (lowest level)	8.82	3.42	88.97	19.81	3.52	0.26
BR						
BO						
1 (first)	7.23	2.83	84.91	16.89	3.58	0.32
2 (second)	8.16	3.38	90.93	15.26	3.45	0.29
3 (third)	8.05	3.07	89.58	13.82	3.62	0.38
OB						
1 (no)	7.58	2.93	87.12	15.56	3.56	0.31
2 (yes)	8.12	3.60	89.66	18.30	3.52	0.33
OS						
1 (no)	7.64	3.05	87.15	16.54	3.55	0.31
2 (yes)	7.94	3.23	89.66	18.30	3.58	0.33
HS						
CTGY						
1 (no)	6.75	2.36	85.69	16.87	3.58	0.30
2 (yes)	8.05	3.25	88.40	15.90	3.55	0.32
TH						
1 (apartment housing block)	7.58	3.41	87.25	16.44	3.55	0.31
2 (apartment in a detached house)	7.74	2.75	88.21	16.65	3.57	0.31
3 (single storey)	8.00	2.64	86.27	15.04	3.58	0.34
4 (villa)	7.65	3.16	88.91	16.46	3.52	0.28
PR						
APM						
1 (younger)	6.60	1.89	76.11	25.03	3.64	0.51
2 (same age)	7.79	3.34	88.12	15.66	3.51	0.30
3 (older)	7.69	2.75	88.54	15.17	3.62	0.33
SPP						
1 (opposite sex)	8.14	2.73	89.97	12.74	3.45	0.24
2 (same sex)	7.62	3.14	87.28	16.68	3.57	0.32
IP						
1 (no)	5.76	1.19	84.80	10.98	3.57	0.19
2 (yes)	7.76	3.11	87.76	16.33	3.55	0.32
EP						
GL						
1 (only in home)	6.58	2.40	82.96	12.85	3.62	0.32
2 (in the home garden)	7.05	2.58	83.77	16.90	3.61	0.31
3 (in the city block)	7.92	2.96	88.96	16.03	3.53	0.33
4 (in the neighbourhood)	10.23	4.70	100.64	13.23	3.38	0.24
5 (free to play anywhere)	9.88	0.01	90.60	0.01	3.65	0.01
RTMF						
1 (mother)	7.78	3.09	88.61	16.25	3.54	0.32
2 (father)	7.19	3.01	81.78	14.60	3.62	0.29
TT						
1 (passive toys)	7.43	2.84	86.36	15.45	3.60	0.33
2 (active toys)	8.23	3.49	90.30	17.32	3.46	0.25
Sum of skinfolds (mm)	M = 40.75; SD = 18.34					

Note: SES = Socio-economic status; LME = level of mother’s education; LFE = level of father’s education; FO = father occupation; MO = Mother’s occupation; BR = sibling characteristics/brotherhood relationship; BO = birth order; OB = having an older brother(s); OS = having an older sister(s); HS = house/living space; TH = type of housing; CTGY = existence near the house of a courtyard, terrace, garden, or yard where the child can play; PR = child’s interaction with peers; APP = age of preferred playmates; SPP = sex of preferred playmates; IP = interaction with peers out of school; EP = educational practices within the family; GL = geographical limit of child’s play in relation to household; TT = type of toys; RTMF = relative time spent by each parent with the child.

## Data Availability

The data presented in this study are available on request from the corresponding author. The data are not publicly available due to privacy concerns.
